# An automated retrospective VAE-surveillance tool for future quality improvement studies

**DOI:** 10.1038/s41598-021-01402-3

**Published:** 2021-11-15

**Authors:** Oliver Wolffers, Martin Faltys, Janos Thomann, Stephan M. Jakob, Jonas Marschall, Tobias M. Merz, Rami Sommerstein

**Affiliations:** 1 Department of Infectious Diseases, Bern University Hospital, University of Bern, Bern, Switzerland; 2 Department of Internal Medicine, Bern University Hospital, University of Bern, Bern, Switzerland; 3 Department of Intensive Care Medicine, Bern University Hospital, University of Bern, Bern, Switzerland; 4 Cardiovascular Intensive Care Unit, Auckland City Hospital, Auckland, New Zealand; 5 Department of Health Sciences and Medicine, St. Anna Hospital, University of Lucerne, Lucerne, Switzerland

**Keywords:** Bacterial infection, Respiratory signs and symptoms, Preventive medicine

## Abstract

Ventilator-associated pneumonia (VAP) is a frequent complication of mechanical ventilation and is associated with substantial morbidity and mortality. Accurate diagnosis of VAP relies in part on subjective diagnostic criteria. Surveillance according to ventilator-associated event (VAE) criteria may allow quick and objective benchmarking. Our objective was to create an automated surveillance tool for VAE tiers I and II on a large data collection, evaluate its diagnostic accuracy and retrospectively determine the yearly baseline VAE incidence. We included all consecutive intensive care unit admissions of patients with mechanical ventilation at Bern University Hospital, a tertiary referral center, from January 2008 to July 2016. Data was automatically extracted from the patient data management system and automatically processed. We created and implemented an application able to automatically analyze respiratory and relevant medication data according to the Centers for Disease Control protocol for VAE-surveillance. In a subset of patients, we compared the accuracy of automated VAE surveillance according to CDC criteria to a gold standard (a composite of automated and manual evaluation with mediation for discrepancies) and evaluated the evolution of the baseline incidence. The study included 22′442 ventilated admissions with a total of 37′221 ventilator days. 592 ventilator-associated events (tier I) occurred; of these 194 (34%) were of potentially infectious origin (tier II). In our validation sample, automated surveillance had a sensitivity of 98% and specificity of 100% in detecting VAE compared to the gold standard. The yearly VAE incidence rate ranged from 10.1–22.1 per 1000 device days and trend showed a decrease in the yearly incidence rate ratio of 0.96 (95% CI, 0.93–1.00, p = 0.03). This study demonstrated that automated VAE detection is feasible, accurate and reliable and may be applied on a large, retrospective sample and provided insight into long-term institutional VAE incidences. The surveillance tool can be extended to other centres and provides VAE incidences for performing quality control and intervention studies.

## Introduction

### Background/rationale

Ventilator-associated pneumonia (VAP) is a frequent healthcare-associated infection^1^ with high crude and attributable mortality rates^2–4^. Clinical criteria to diagnose VAP lack sensitivity and specificity when compared to autopsy reports^5^ and were shown to be associated with substantial inter-observer variability^6^, making the diagnosis of VAP difficult and not uniformly defined. In 2013 the United States’ Centers for Disease Control and Prevention (CDC) released its new surveillance protocol for ventilator-associated events (VAE) in order to address the above-mentioned problems^7^. The protocol consists of three tiers with a stepwise approach: Tier I—Ventilator-associated condition (VAC)—is defined as worsening oxygenation after a baseline period of two days with stable or decreasing positive end-expiratory pressure (PEEP) or fraction of inspired O_2_ (FiO_2_). Tier II—infectious ventilator-associated condition (IVAC)—consists of all VAC with a newly administered antimicrobial agent and either an abnormal white blood cell count (WBC) or body temperature outside the normal range. Tier III represents possible ventilator-associated pneumonia (PVAP), comprising IVAC with either purulent respiratory secretions or detection of a defined set of pulmonary pathogens. Screening for VAC has shown non-inferior sensitivity and specificity for diagnosing VAP^8^ when compared to classical criteria like national healthcare safety network PNEU criteria^9^ requiring clinical signs of pulmonary infection, imaging and pathological laboratory results. Classification according to VAE-criteria was reported to be a superior predictor of outcomes^10^.

Since its introduction, several implementations of automation have been described in the United States and the Netherlands^11–13^. VAE reporting has become mandatory in parts of the United States, where an economic incentive to streamline VAE surveillance was created. To our knowledge, the VAE protocol for VAP surveillance has not yet been formally implemented outside of the US. Our aim was therefore to create a fully automated VAE surveillance tool for the first two tiers of the CDC VAE surveillance protocol and assess its diagnostic accuracy. Furthermore, we aimed to determine whether the retrospectively identified cases indicated a dynamic in the VAE incidences from 2008 to 2016 and to establish a baseline incidence.

## Methods

### Study design

Creation of a fully automated surveillance tool and retrospective analysis of VAE cases.

### Setting

This validation study of a retrospective surveillance was carried out at Bern University Hospital, a Swiss tertiary care hospital with 950 beds and 4500 ICU admissions per year, most of them being mechanically ventilated for at least a short period. The ICU is organised as an interdisciplinary 37-bed unit admitting all adult surgical and medical patients. Clinical data was prospectively collected in the unit’s electronic patient data management system (PDMS; GE Centricity Critical Care, General Electrics, Helsinki, Finland). This PDMS provides a versatile information management tools for the intensive care unit. It handles the fully automated collection of equipment data, and ensures reliable treatment documentation at the bedside.

### Participants

All patient admissions to the ICU between January 2008 and July 2016 with at least one record of mechanical ventilation were included in the study.

### Variables

The primary outcome variable was the occurrence of a VAE (Tier I or II; according to CDC criteria^7^).

In accordance with Shenoy et al.^14^ we defined our “gold standard” for VAE as a composite of detected cases either identified by manual and/or automated surveillance^14^ with a formalized resolution of differences. In case of disagreement between the two methods, presence or absence of VAE was determined by an independent senior infection prevention physiciant.

Criteria for VAE tiers I and II were defined by the CDC protocol and included minimal daily FiO_2_, minimal daily PEEP, minimal and maximal daily body temperature; minimal and maximal daily WBC and new antimicrobial agent administration.

Manual surveillance was performed by an unbiased member of the study team on plotted data of the criterion variables, an example can be seen in supplementary Fig. 2.

The number of ventilated patients per day was defined as presence of mechanical ventilation at noon of a given day (defined by a two-hour window around 12:00).

### Data source/measurements

Criteria variables were obtained via a structured query from the ICU’s PDMS repository. Automatically measured values were recorded every two minutes (e.g., FiO_2_) to 15 min (e.g. PEEP), manually documented values according to clinical necessity, but at least once per 8 h shift (e.g., tympanic temperature). Administration of antimicrobial therapy and the corresponding dose was also extracted automatically from the PDMS. The source of the information per criterion variable varied over the years and some criteria had multiple concurrent sources for one individual (e.g., temperature from axillary and tympanic measurement). Data source and measurements are summarised in Supplementary Table 1. The protocol and source code documenting how the raw data was cleared, handled, and evaluated for VAEs is available online^15^.

We created a software called “Event Reader” (C#, .Net version 4.5 with Windows Presentation Foundation [WPF] front end) to process the criteria variables into one single daily value per variable, as required to calculate VAEs. There was no distinct variable signalling mechanical ventilation over the entire observation span. The period of mechanical ventilation was thus derived from the continuous availability (one measurement per 6 min window) of end-tidal CO_2_ (etCO_2_) measurements in ventilated patients. Only FiO_2_ and PEEP values recorded during this period of time were used in our analysis. Data cleaning was performed at this step as described below. The resulting daily variables per patients were then automatically entered into a Microsoft Access database (Microsoft, Redmond WA, U.S.).

To calculate VAEs, the same software (Event Reader) applied a slightly adapted algorithm (as described before^16^) from the daily variables of the Access database (Supplementary Fig. 1).

### Data cleaning and sensitivity analysis

The recorded respiratory values contained outlying values that likely were incorrect measurements or unintended deviations (example visualized in Supplementary Fig. 2). The CDC’s rules define the minimum respiratory parameters per day as the value kept stable over at least one hour or if no such value was available to use the lowest recorded value. As previously suggested^12^, we corrected for low outliers excluding a predetermined percentage of the lowest values, since only they influence the recorded lowest daily value. A sensitivity analysis was carried out to ascertain the correct threshold (comparing exclusion of the lowest 5% and 10% to no exclusion).

Measured PEEP and especially FiO_2_ values tended to oscillate around the set value. We anticipated an issue in instances where the measured value such as FiO_2_ did not correspond exactly to the set value, e.g. a FiO_2_ that was set to 60% but being measured as 58%. To correct for this potential signal noise, we investigated different tolerances to the standard CDC’s predefined threshold of worsening of ventilation (increase of 3 cm H_2_O PEEP and/or 20% FiO_2_). We carried out a sensitivity analysis comparing threshold tolerances of 0%, 10% and 20%, respectively.

Confounding FiO_2_ and PEEP values from non-invasive ventilation were excluded from VAE screening because a period of assisted ventilation longer than 3 calendar days was needed to qualify for VAE. Institutional policy prohibits such long periods of non-invasive ventilation.

### Study size

Study size was determined by the number of patients with at least one episode of mechanical ventilation during the study period.

### Quantitative variables

FiO_2_ values below 0.30 were set to 0.30, PEEP-values below 5 cmH_2_O to 5. Temperature values below 35 °C and above 42 °C were excluded.

### Statistical methods

We applied confusion matrices to obtain the sensitivity, specificity, and positive and negative predictive (PPV and NPV) values of the VAEs determined by automated surveillance compared to the gold standard.

We chose three samples to compare the automated surveillance’s performance:

(1) a convenience sample of 131 admissions with prolonged mechanical ventilation periods of 21–27 days to determine sensitivity and specificity regarding VAC detection, (2) a random (sample_n, *dplyr* package in R), sample of 100 VAC out of all VAC as identified by the algorithm to determine primarily specificity compared to the composite gold standard and (3) a random sample of 100 verified (according to the gold standard) VAC to evaluate the algorithm’s sensitivity and specificity for detection of IVAC. Suppl. Figure 4 summarizes the selection of different validation samples.

Sensitivity analysis for threshold augmentation and outlier cutoffs were carried out using the same convenience sample.

The yearly VAE incidence rate was defined as the number of VAEs per 1000 ventilator days and analysed per year for the surveillance period. Potential trends in the rate were evaluated by a Poisson regression model.

### Ethics approval and consent to participate

According to Swiss federal law, healthcare-associated infection surveillance is considered a quality improvement project and therefore exempt from ethical approval. This study was carried out in accordance with relevant guidelines and regulations.

## Results

### Creation and validation of a fully automated VAE surveillance

**Figure 1:**
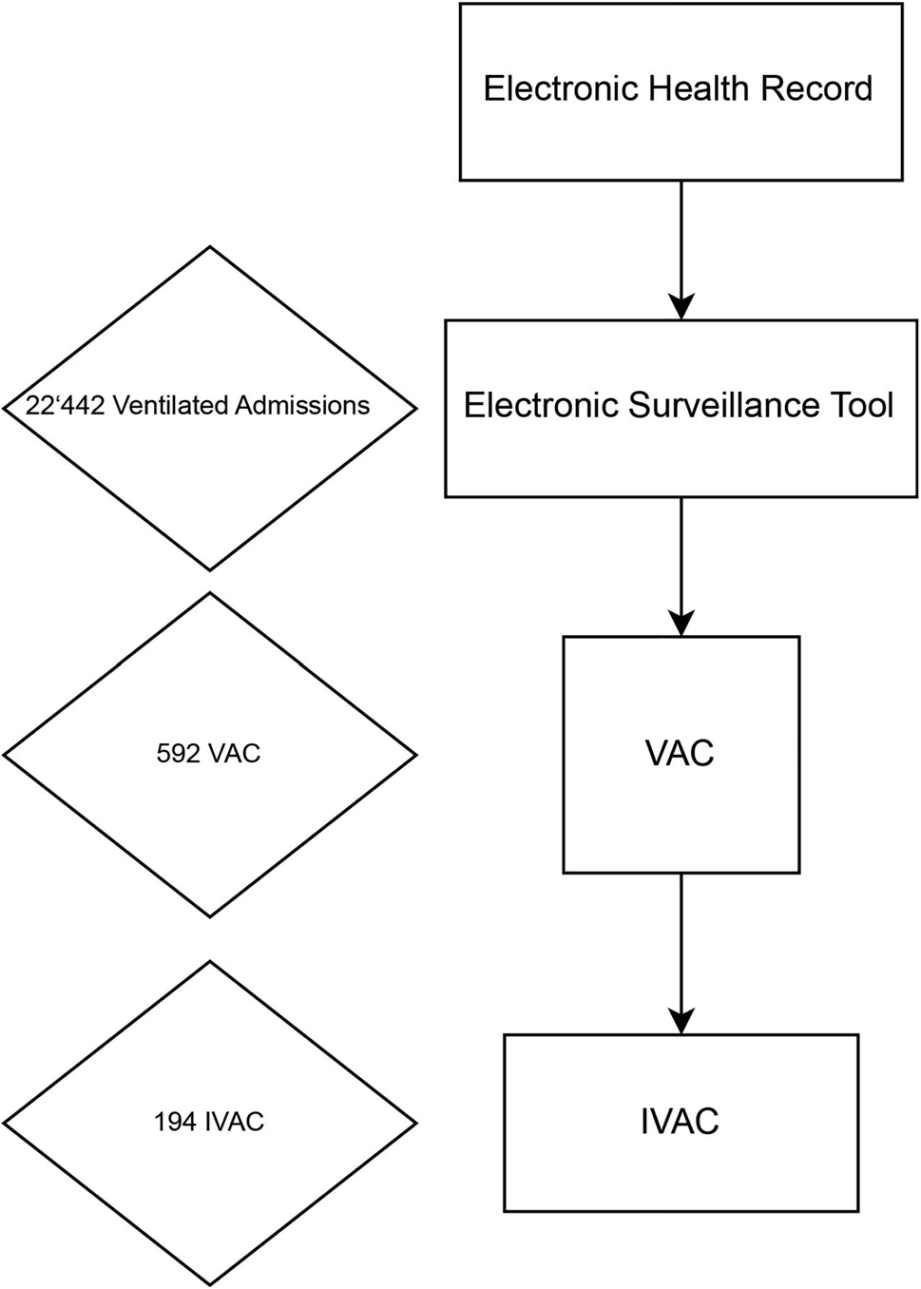
Flowchart showing information flow and information processing. VAC Ventilator associated condition; IVAC Infectious ventilator associated condition.

During the observation period from January 2008 to May 2016, 22′442 admissions of ventilated patients with a total of 37′221 ventilator days (Flowchart, Fig. 1) occurred and were included in the analysis. Patients were ventilated for a median of 1 day (IQR 1–3). Automated surveillance detected 592 VAE in 2.5% of all the patients. This corresponds to 15.9 VAEs/1000 ventilator days (95% CI, 14.7—17.2). Note that as per the CDC protocol, we included all ventilated patients in the denominator, even those not ventilated for 4 calendar days which per definition could not qualify for VAE. Of the VACs, 205 (35%) were IVACs.

Automated surveillance was faster than manual surveillance. The entire observation period with around 22′400 admissions was screened automatically in around 12 h using a standard laptop computer. Conversely, screening a single admission using manual surveillance required an experienced observer around 2 min per case which would have resulted in around 800 h for manual surveillance of the entire dataset.

### Evaluation of diagnostic accuracy

In the convenience sample of the 131 patients with ventilation of 21 to 27 days, the sensitivity of automated surveillance with the Event Reader compared to the gold standard demonstrated a sensitivity of 98%, a specificity of 100%, an NPV 99% and a PPV of 100% (Table 1).

**Table 1:** Analysis for sensitivity and specificity comparing VAC-surveillance to the gold standard. Cut-off indicates the percentage setting of the lowest outliers being excluded at import. Threshold shows different tolerance threshold settings for including respiratory deterioration as significant according to VAE rules.

Mode	Cut-off	Threshold	TP	FP	FN	TN	Total	Sensitivity	Specificity
Auto	5%	10%	42	0	1	89	132	0.98	1
Auto	0%	10%	27	8	16	85	136	0.63	0.91
Auto	10%	10%	39	4	4	87	134	0.91	0.96
Auto	5%	0%	42	0	1	89	132	0.98	1
Auto	5%	10%	42	0	1	89	132	0.98	1
Auto	5%	20%	42	0	1	89	132	0.98	1
Manual	Visual	Visual	32	4	10	86	132	0.76	0.96

TP True Positive; FP False Positive; FN False Negative; TN True Negative; VAC Ventilator Associated Condition.

Conversely, manual surveillance against the gold standard for determination of VAC (Tier I) was 76%, the specificity 96%, the positive predictive value (PPV) 89%, and the negative predictive value (NPV) 90%.

For the VAC validation step with the randomly selected 100 VAEs (as determined by the reader), specificity was 99% and PPV 99% when compared to the gold standard.

In a randomly generated sample of 100 VAC (Tier II), the sensitivity of automated surveillance for IVAC detection was 100% and the specificity 100% compared to the gold standard. Manual surveillance yielded a sensitivity of 88% and a specificity of 97% (Table 2).

**Table 2:** Analysis for sensitivity and specificity comparing IVAC surveillance to the gold standard surveillance.

Setting	TP	FP	FN	TN	Total	Sensitivity	Specificity	PPV	NPV
Automated	35	0	0	65	100	1	1	1	1
Manual	29	2	4	65	100	0.88	0.97	0.94	0.94

TP True Positive; FP False Positive; FN False Negative; TN True Negative; IVAC Infectious Ventilator Associated Condition.

The additional analysis for evaluating the influence of low outliers showed the algorithms best sensitivity for a cut-off value of 5% (98%), while cut-offs of 0% and 10% had a sensitivity of 63% and 91%, respectively (Table 1) when compared to the gold standard. Best specificity was also reached with the 5% cut-off (100%). The additional analysis regarding FiO_2_/PEEP increase tolerance thresholds yielded no influence on sensitivity/specificity (Table 1).

### Determination of yearly VAE incidence

**Figure 2:**
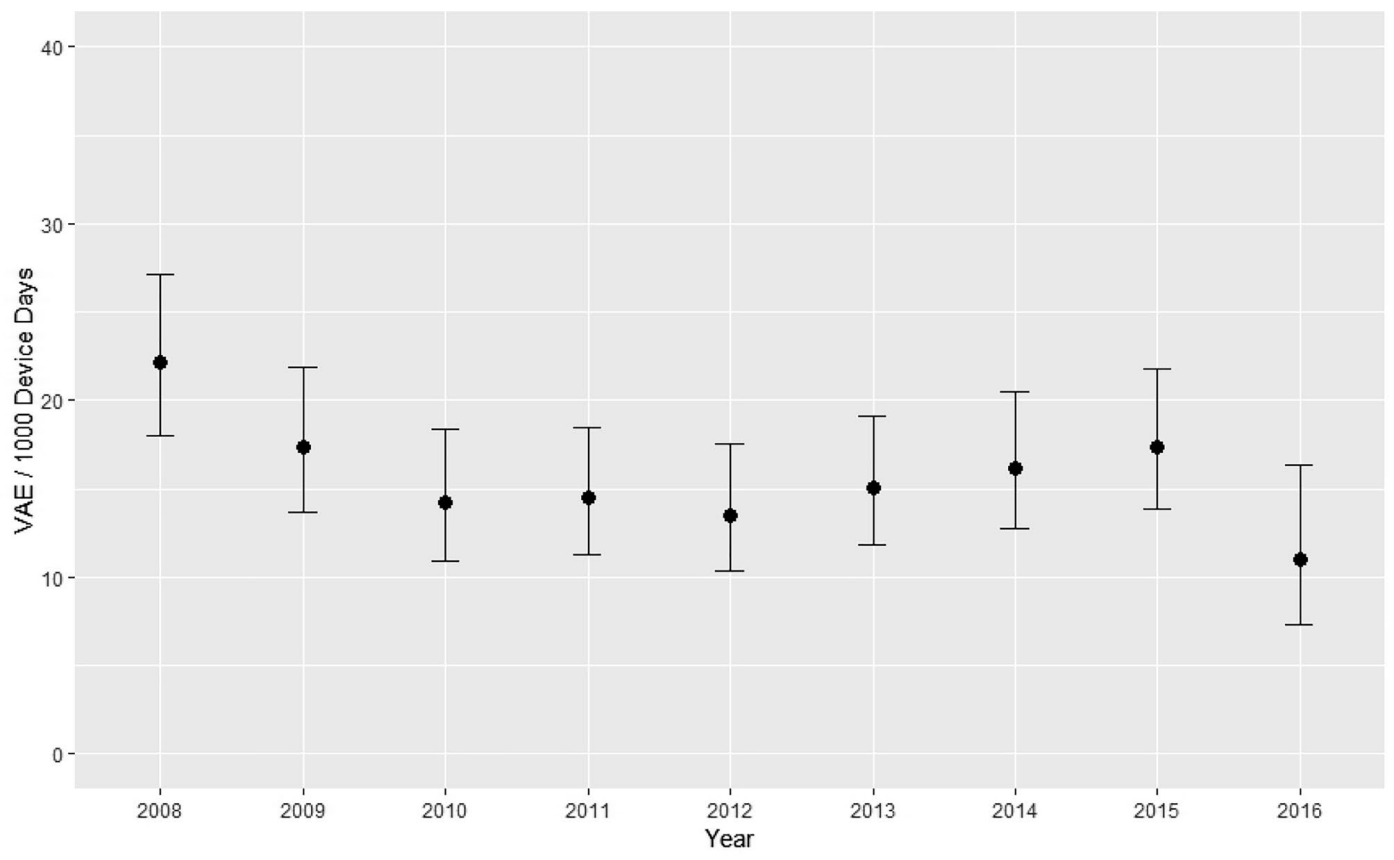
Incidence of VAE from 2008 to 2016. VAE Ventilator associated events.

The yearly VAE incidence rate per 1000 device days is shown in Fig. 2 and Table 3. It ranged from 22.1/1000 ventilator days (95% CI 17.4–26.3) in 2008 to 10.1/1000 (CI 7.0–15.8) 2016. Over the entire observation period there was an incidence rate of 15.9/1000 ventilator days (95% CI 14.7–17.2).

**Table 3:** Ventilator associated events per year.

Year	VAE	Device days	Ratio	Lower 95% CI	Upper 95% CI
2008	94	4251	22.1	18	27.1
2009	71	4100	17.3	13.6	21.9
2010	60	4223	14.2	11	18.4
2011	67	4634	14.5	11.3	18.4
2012	58	4303	13.5	10.3	17.5
2013	69	4582	15.1	11.8	19.1
2014	70	4333	16.2	12.7	20.5
2015	77	4430	17.4	13.8	21.8
2016	26	2365	11	7.34	16.3

VAE Ventilator associated events; Device days Total number of ventilated patient days; CI Confidence Interval.

According to our Poisson regression model, there was a significant yearly incidence decrease at a rate ratio of 0.96 (95% CI, 0.93—1.00, p = 0.03). When excluding 2008 and 2016 which had considerably less records available, the yearly incidence rate ratio showed no statistically significant change over the observation period (1.01; 95% CI, 0.97–1.06, p = 0.61).

## Discussion

We created and validated an automated VAE surveillance and retrospectively evaluated its diagnostic accuracy. Automated surveillance was faster and more reliable than manual surveillance for the detection of VAE. The incidence rate ratio showed a slight but statistically significant reduction over the observation period. To our knowledge, our results represent by number of enrolled patients the largest dataset employed to date^17^. This implementation was feasible even though the variable “mechanical ventilation” itself—a prerequisite for VAE—was not available in the PDMS. The tool can readily be adapted to other data platforms and their respective electronic health records. Our findings are in accordance with previous studies, albeit with our study having a significantly larger sample size concerning the observation period^11–13,16^.

The incidence rate ratio showed a slight but significant decrease over the observation period that was mostly caused by the first and last year (2008 and 2016) in the observation period, which did have a significantly lower number of records. When looking at the period from 2009 to 2015 there was no change in the incidence rate ratio. We acknowledge that the long term incidence rate depends on the validation of a small subset. Therefore, 492/592 VAEs have not been confirmed by manual surveillance and we cannot exclude that sensitivity and predictive value for all VAEs may differ from the validated subset.

Most manual detection errors lay in mere oversights while screening the available data, resulting in error. Automated surveillance was not only more precise but also much quicker, even when using standard electronic equipment.

The sensitivity analysis for import cut-offs confirmed earlier publications that showed a superiority of using a percentage cut-off at import^12^. Sensitivity analysis to determine the amount of lowest outlying values ignored at import showed a superiority of using a 5% cut-off compared to using 0% cut-off where even small artefacts distorted the daily lowest recorded values. Using a 10% cut-off turned out to ignore too many relevant data points. Sensitivity analysis for the tolerance of respiratory deterioration values revealed no difference in sensitivity when varying the tolerance between 0 and 20% for the examined sample with no additional VAC being found. Thus, using cut-offs to accommodate for measured values oscillating around a set point showed to be less important.

Internal validity proved to be excellent as is shown by the very high sensitivity and specificity. The stability of our results over the years also may indicate a considerable robustness of our approach. Furthermore, our calculated event rate per 1000 ventilator days was in the same range as the 10/1000 ventilator days as estimated by Klein et. al^12^.

As was shown previously^18,19^, automated VAE surveillance can be liable to being gamed in order to reduce the incidence of VAC and thus reduce the overall prevalence of VAE. Since VAE has so far not been used as a metric we do not think this applies to this dataset which actually reflects the true incidence over the observation period.

This automated surveillance comes at a price however, as was also shown in other studies^20^: ongoing surveillance requires continuous maintenance in order to keep it operational with prospective software changes.

### Limitations

Our study has several limitations. First, an inherent limitation was that non-invasive ventilation was also identified as mechanical ventilation as the database does not contain a variable over the entire observation period to reliably indicate the presence of an artificial airway. It is important to note that per institutional regulations, non-invasive ventilation is rarely maintained for > 48 h and therefore we are confident that all VAE did occur under invasive mechanical ventilation. While this limitation affects denominator data, it did not confound identification of VAE. If institutional regulations concerning the usual duration of non-invasive ventilation were different, this would lead to an overestimation of VAE incidence. Second, because of overall rather low prevalence of VAE, we used a small sample of patients who were ventilated for more than 22 days, thus yielding samples with more complicated cases to evaluate. This may have negatively impacted the performance of manual surveillance, thus the advantage of automated performance compared to manual surveillance might be less important in a more representative sample with cases of shorter duration. We differed in our approach from the CDC guidelines considering the definition of the lowest recorded respiratory value as we used the lowest recorded value after excluding the lowest 5%, a comparison with the standard approach using the lowest hourly setting was considered less feasible for technical reasons.

### Outlook

As we have shown, an implementation of a retrospective VAE surveillance tool is feasible, even when the data management system at the local ICU was initially not set up for this purpose.We believe that this approach can be taken in hospitals using similar patient data management systems and only at modest cost and effort. This would permit the retrospective establishment of a baseline VAE incidence. Quality control and intervention studies should be undertaken to investigate changes, possibly resulting in a lower VAE incidence. The artefact removal rate may depend on the used equipment in the respective ICU. Close coordination between infection preventionists and information technology specialists is necessary in order to achieve a smooth integration. Specifically, the information infrastructure of this hospital was not designed for easy data extraction and computation in the context of research projects. Automated surveillance requires maintenance as well, especially in preserving a robust data structure despite changes in hard- or software.

## Conclusion

We created a VAE surveillance which is ready to be implemented in an ICU with a patient data management system using high sampling frequency. As we have shown in our samples, automated surveillance is more sensitive and more specific than manual surveillance, thus enabling transition to more comprehensive and more reliable VAE surveillance programs. This study demonstrates that automated VAE detection is feasible on a large, retrospective sample. The tool could readily be expanded to further centres. It can be used as routine screening for VAE, thus establishing a baseline incidence for future quality control and intervention studies.

## Supplementary Information

Supplementary Information 1.

Supplementary Information 2.

## Data Availability

The datasets generated and/or analysed during the current study are not publicly available due to institutional privacy guidelines but are available from the corresponding author on reasonable request.
