# Supplementary material for: An automated retrospective VAE-surveillance tool for future quality improvement studies
Source: Sci Rep. 2021 Nov 15;11:22264. doi: 10.1038/s41598-021-01402-3 (PMC8593155; doi:10.1038/s41598-021-01402-3)
Supplement: Supplementary file 1 — Supplementary Information 1. [file 41598_2021_1402_MOESM1_ESM.docx]

Supplementary Table 1

| **Legend** |  |
| --- | --- |
| FiO2 | Fraction of inspired O2 |
| PEEP | Positive End-expiratory pressure |
| EtCO2 | End-tidal CO2 |
| WBC Count | White blood cell count |
| PDMS | Patient data management system |

| **Value** | **Point of Measurement** | **Frequency** | **Number of different variables** |
| --- | --- | --- | --- |
| FiO2 (%) | Ventilator measurement | Every 2 minutes | 1 |
| PEEP (mmHg) | Ventilator measurement | Every 15 minutes or when setting changes | 1 |
| EtCO2 (mmHg) | Capnographic sensor | Every 2 minutes | 3 |
| Temperature (°C) | Nasal Temperature  Core Temperature  Tympanic Temperature | Every 2 minutes for continuous measurements or as measured | 4 |
| WBC Count (G/L) | Automated report from hematology laboratory to the PDMS | As measured, usually 1/day | 1 |
| Antimicrobial Drugs | As recorded in the PDMS | Cumulative daily dose for every antimicrobial agent administered |  |

Supplementary Table 1 Showing the relevant variables and the frequency of their recording

| **Legend** |  |
| --- | --- |
| FiO2 | Fraction of inspired O2 |
| PEEP | Positive End-expiratory pressure |
| EtCO2 | End-tidal CO2 |
| WBC Count | White blood cell count |
| PDMS | Patient data management system |


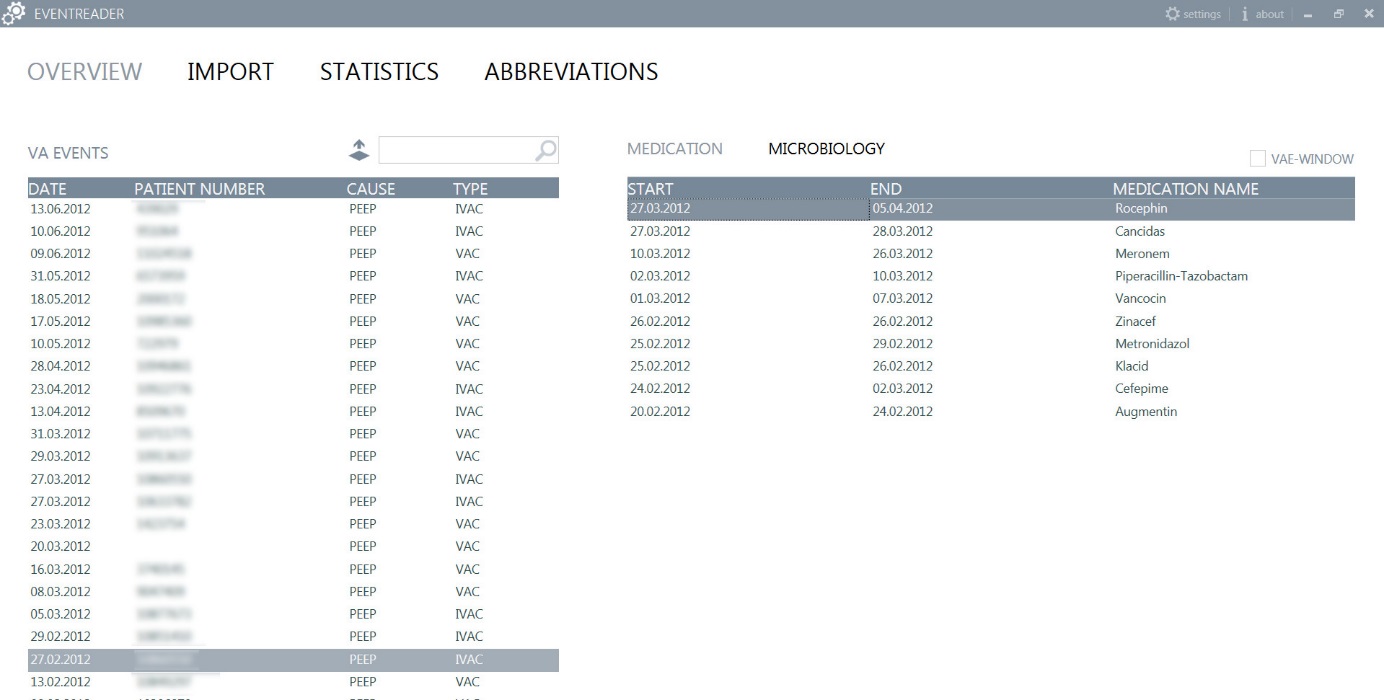


Supplementary Figure 1

| **Legend** |  |
| --- | --- |
| VAE | Ventilator associated events |
| FiO_2_ | Fraction of inspired O2 |
| PEEP | Positive end expiratory pressure |

Supplementary Figure 1

A Screenshot of the database interface, on the left hand side are the patient cases, for the selected one are the antimicrobial administrations displayed, either for the entire stay or if wanted only the administrations within the VAE-window


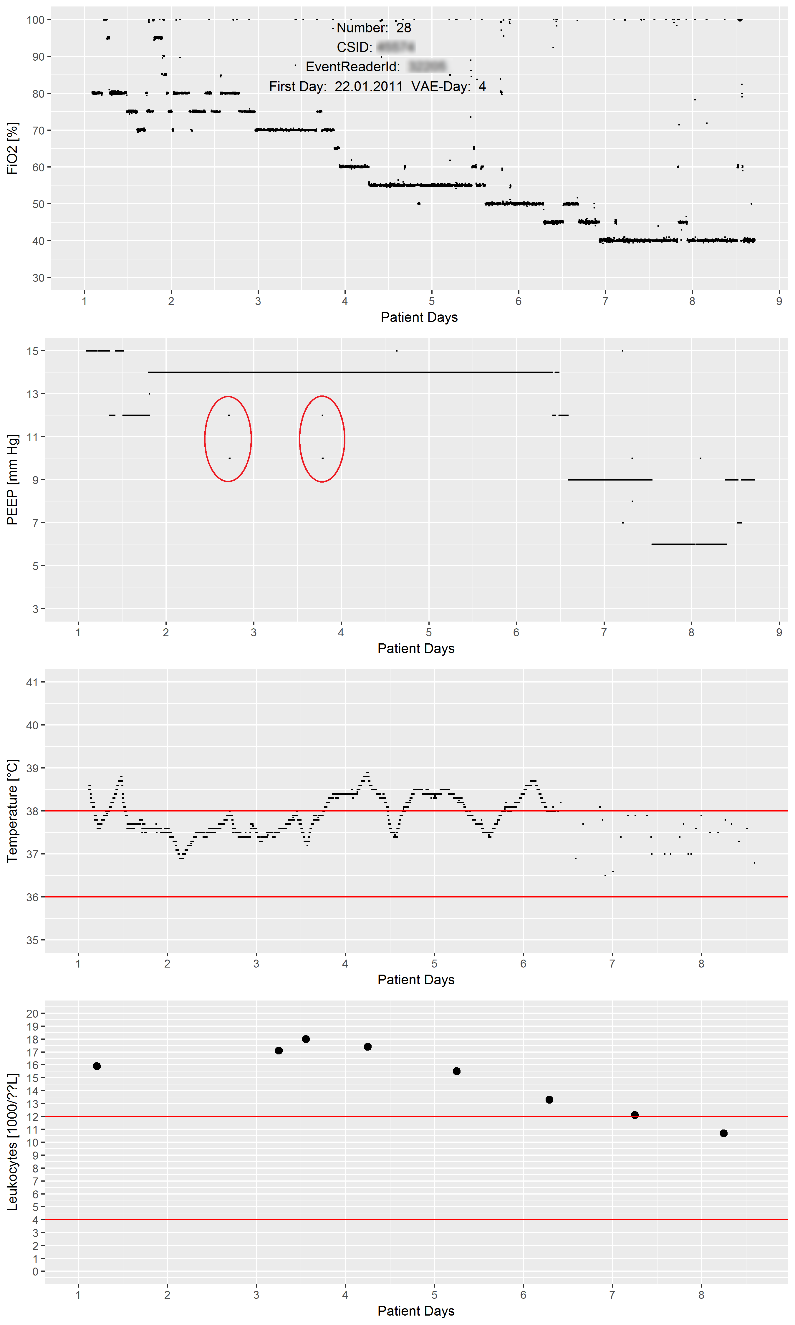


Supplementary Figure 2

Supplementary Figure 2

Graphical representation of the raw data, used to carry out the manual part of surveillance. A plot of one ventilated patient showing the patients entire stay with FiO_2_, PEEP, Temperature and WBC from top to bottom, the red lines indicate reference values for the upper and lower norm. Notice the red circles indicating two recorded low outliers on day 2 and day 3. These data points obviously do not represent the actual respiratory situation of the patient and were classified as artefacts. According to our protocol, we disregarded the lowest 5% of recorded respiratory values (PEEP and FiO_2_). As can be seen, recorded FiO_2_ values tended to oscillate around the set value. To filter out this signal noise we used a tolerance added to the standard CDC’s predefined threshold of worsening of ventilation (increased of 3cm H_2_O PEEP and/or 20% FiO_2_), thus reducing the needed increase by the threshold’s percentage. We carried out a sensitivity analysis comparing threshold augmentations of 5%, 10% and 20% to the base value.

| **Legend** |  |
| --- | --- |
| VAE | Ventilator associated events |
| FiO_2_ | Fraction of inspired O2 |
| PEEP | Positive end expiratory pressure |


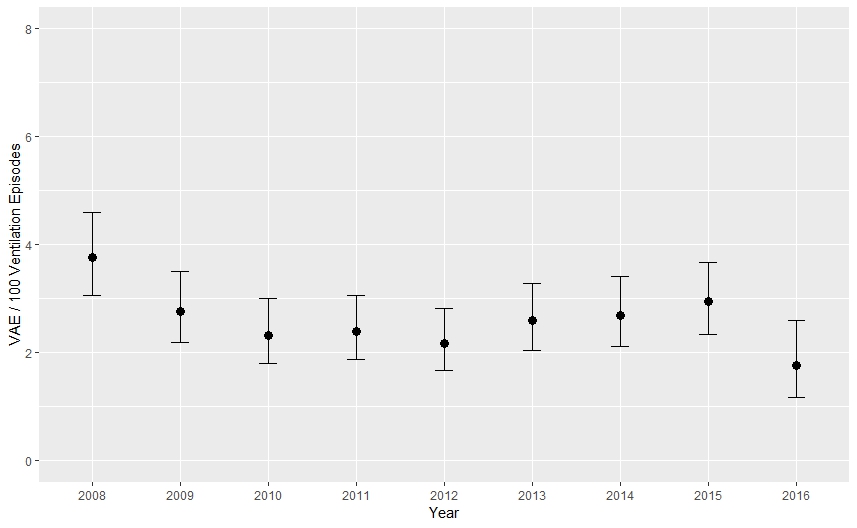


Supplementary Figure 3

VAEs/100 ventilation episodes

Supplementary Figure 3

Graphical representation of the incidence of VAE and its confidence intervals per 100 episodes of mechanical ventilation

| **Legend** |  |
| --- | --- |
| VAE | Ventilator Associated Events |


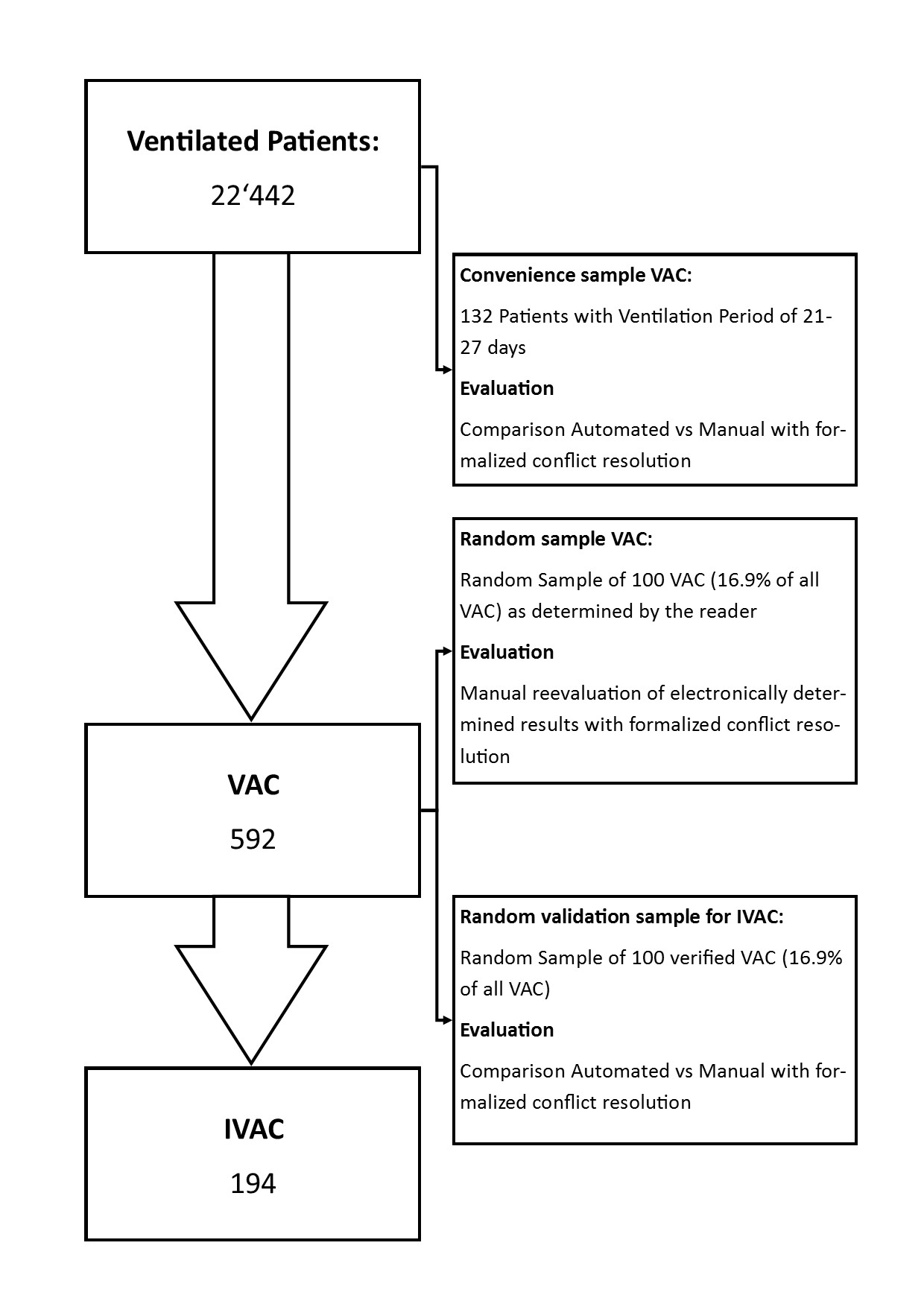


Supplementary Figure 4

Supplementary Figure 4

Flowchart depicting the selection of the different populations used for validation of our surveillance algorithm.

| **Legend** |  |
| --- | --- |
| VAE | Ventilator associated events |
| VAC | Ventilator Associated Condition |
| IVAC | Infectious Ventilator Associated Condition |
