# Supplementary material for: An automated retrospective VAE-surveillance tool for future quality improvement studies
Source: Sci Rep. 2021 Nov 15;11:22264. doi: 10.1038/s41598-021-01402-3 (PMC8593155; doi:10.1038/s41598-021-01402-3)
Supplement: Supplementary file 2 — Supplementary Information 2. [file 41598_2021_1402_MOESM2_ESM.docx]

**Supplementary script files**

Identifying Validation Population.R

Used to find the population for the validation convenience sample and creating the visual representation of the data

EventsAndDeviceDays.R

Used to calculate VAE/Device Days

EventsPerHEpisodes_export.R

Used to calculate VAE/100 Ventilation Episodes

ValidateIVAC.R

Used to create the random validation sample for IVAC-validation

**Supplementary script files**

Identifying Validation Population.R

# This script allows for the creation of a short subgroup of a specific length of stay in ICU

# This subgroup is then plotted in a 1x4 Plot with PEEP, FiO2, Leuk and Temp in order to

# compare the human identification performance to the one of the EventReader

library(tidyverse)

library(lubridate)

library(dplyr)

library(readr)

library(ggplot2)

library(plyr)

source("SOURCE")

setwd("SOURCE")

#imports all relevant Datasets from the wd

CalculationSets <- read_delim("CalculationSets.csv", delim = ";")

Events <- read_delim("Events.csv", delim = ";")

Medication <- read_delim("Medications.csv", delim = ";")

Patients <- read_delim("Patients.csv", delim = ";")

relCalculationsets <- subset(CalculationSets, CalculationSets$IsIntubatedAtNoon=="WAHR")

#creates a list of all patients, how long they stayed and plots the frequency of each duration

StayDuration<-aggregate(Date~Patient_Id, data=relCalculationsets, function(x) length(unique(x)))

names(StayDuration)[2]<-"Duration"

ICUstays <- count(StayDuration, "Duration")

ggplot(ICUstays, aes(x = ICUstays$Duration, y = ICUstays$freq)) +

geom_point()

median(StayDuration$Duration)

PersonOfInterest <- subset(StayDuration, (StayDuration$Duration<27 & StayDuration$Duration > 22))

#Now we need to match the various Id-numbers across different tables in order to identify our patients

LookUpTable <- merge(PersonOfInterest,Patients, by.x = "Patient_Id", by.y = "Id")

names(LookUpTable)[1] <- "EventReaderId"

#Import of the big dataset, we'll drop most values after the import tho

setwd("SOURCE")

dat <- read_delim("vap_ventilated.csv",

",", escape_double = FALSE, trim_ws = TRUE)

#Renaming columns according to standard practice

colnames(dat) <- c("X1","PatientID", "Datetime", "fio2_m", "peep_m", "MechanicalVentilation", "Leuk", "TempCentral")

# selects only the patients from the lookuptable

dat<-merge(dat,LookUpTable, by.x = "PatientID", by.y = "CaseId")

#dat<- subset(dat, PatientID == ID)

#eliminating impossible values, dropping ventilation values that are flagged mechanical_venilation = FALSE

dat$peep_m[dat$peep_m<0] <- NA

dat$TempCentral[dat$TempCentral<0]<-NA

dat$TempCentral[dat$TempCentral>41]<- 41

dat$Leuk[dat$Leuk >20] <- 20

dat$peep_m[dat$MechanicalVentilation == "False"]<-NA

dat$peep_m[dat$peep_m <3]<-3

dat$fio2_m[dat$MechanicalVentilation== "False"]<- NA

dat$fio2_m[dat$fio2_m<30] <- 30

dat <- dat[order(dat$Datetime),]

subdat <- dat

#determine minutes

#creates several auxiliary variables in order to determine the right minute value for each datapoint

b1<-as.Date("2006-01-01")

subdat$day<- floor_date(subdat$Datetime,unit="day")

subdat$diff<-difftime(subdat$day,b1,units='days')

subdat$hour = as.numeric(format(as.POSIXct(subdat$Datetime,format="%Y-%m-%d %H:%M:%S"),"%H"))

subdat$min = as.numeric(format(as.POSIXct(subdat$Datetime,format="%Y-%m-%d %H:%M:%S"),"%M"))

subdat$gmin<-subdat$diff*1440+subdat$hour*60+subdat$min

#creates table TempAndLeuk with only Datetime, ID, temperature and leukocyte values

#creates the same timeline auxilliary variables as with the respiratory values

TempAndLeuk<-subset(dat, TempCentral!=""|Leuk!="",

select=c(Datetime, PatientID, TempCentral, Leuk))

TempAndLeuk$day<- floor_date(TempAndLeuk$Datetime,unit="day")

TempAndLeuk$diff<-difftime(TempAndLeuk$day,b1,units='days')

TempAndLeuk$hour = as.numeric(format(as.POSIXct(TempAndLeuk$Datetime,format="%Y-%m-%d %H:%M:%S"),"%H"))

TempAndLeuk$min = as.numeric(format(as.POSIXct(TempAndLeuk$Datetime,format="%Y-%m-%d %H:%M:%S"),"%M"))

TempAndLeuk$gmin<-TempAndLeuk$diff*1440+TempAndLeuk$hour*60+TempAndLeuk$min

for (i in 1:120) {

#pati is a subset of one patient at a time, loops through all patients

pati<-subset(subdat, (PatientID==LookUpTable[i,4]))

#We need to drop all lines without respiratory data for plotting

pati<-subset(pati, pati$MechanicalVentilation==TRUE)

#patiTAL is the same subset for leukocyte and temperature values

patiTal<-subset(TempAndLeuk, (PatientID==LookUpTable[i,4]))

#fl is used to correct the offset minute values, their original zero value is the first measured value of the first patient

fl<-floor(as.numeric(pati$gmin[1])/1440)

#pati$gm is a decimal value of the number of days the patient has been mechanically ventilated

#+1 because the first minute is already on day 1 and not on day 0

pati$gm<-(pati$gmin-1440*fl)/1440+1

patiTal$gm<-(patiTal$gmin-1440*fl)/1440+1

#lastDay is the maximum gm value for this patient. It's mostly used to determine the x-axis

lastDay<- as.numeric(max(pati$gm, na.rm = true))

#annotateposition determines the position of the CSID and PatNumber Label

annotatePosition<-lastDay/2

#tempcentral and other values somehow arent't numeric values

patiTal$TempCentral<- as.numeric(patiTal$TempCentral)

patiTal$Leuk<-as.numeric(patiTal$Leuk)

pati$gm <- as.numeric(pati$gm)

#create a list of all events of the chosen Patient

patiEvents<-subset(Events, Events$Patient_Id==LookUpTable[i,1])

AllEvents <- toString(patiEvents$Date)

#plots fiO2_m

# plotFiO<-ggplot(pati, aes(x=gm, y= pati$fiO2_m))+

# geom_point(size=0.25)+

# scale_y_continuous(breaks=seq(30, 100, 10), limits = c(30, 100))+

# scale_x_continuous(breaks=seq(1,lastDay+2), name="Patient Days")+

# expand_limits(x = 1, y = 0) +

# annotate("text", x=annotatePosition, y=95, label = paste("Number:", i, "\n", "CSID:", LookUpTable[i,4], "\n",

# "EventReaderId:", LookUpTable[i,1]))

plotFiO<-ggplot(pati, aes(x=gm, y= pati$fio2_m))+

geom_point(size=0.25)+

scale_y_continuous(breaks=seq(30, 100, 10), limits = c(30, 100), name="FiO2 [%]")+

scale_x_continuous(breaks=seq(1,lastDay+2), name="Patient Days")+

coord_cartesian(xlim=c(0,lastDay+2), ylim=NULL)+

annotate("text", x=annotatePosition, y=90, label = paste("Number:", i, "\n", "CSID:", LookUpTable[i,4], "\n",

"EventReaderId:", LookUpTable[i,1],

"\n", "First Day: ", format(min(pati$Datetime),"%d-%m-%y %H:%M:%S")))+

annotate("text", x=annotatePosition+2, y=100, label = paste("ReaderEvents: ", AllEvents))

#plots peep_m

plotPeep<-ggplot(pati, aes(x=gm, y= pati$peep_m))+

geom_point(size=0.25)+

scale_y_continuous(breaks=seq(3, 15, 2), limits = c(3, 15), name="PEEP [mm Hg]")+

scale_x_continuous(breaks=seq(1,lastDay+2), name="Patient Days")+

coord_cartesian(xlim=c(0,lastDay+2), ylim=NULL)

#plots Temp

plotTemp<-ggplot(patiTal, aes(gm, TempCentral))+

geom_point(size=0.25)+

geom_hline(yintercept = 38, color="red")+

geom_hline(yintercept = 36, color="red")+

scale_y_continuous(breaks=seq(35, 41, 1), limits = c(35, 41), "Temperature [°C]")+

scale_x_continuous(breaks=seq(0,lastDay+2), name="Patient Days")+

coord_cartesian(xlim=c(0,lastDay+2), ylim=NULL)

#plots Leuk

plotLeuk<-ggplot(patiTal, aes(gm, Leuk))+

geom_point(size=2.55)+

geom_hline(yintercept = 12, color="red")+

geom_hline(yintercept = 4, color="red")+

scale_y_continuous(breaks=seq(0, 20, 1), limits = c(0, 20), name= "Leukocytes [1000/??L]")+

scale_x_continuous(breaks=seq(0,lastDay+2), name="Patient Days")+

coord_cartesian(xlim=c(0,lastDay+2), ylim=NULL)

setwd("SOURCE")

#creates a multiplot out of the two single plots. Uses the custom function multiplot

ggsave(multiplot(plotFiO, plotPeep, plotTemp, plotLeuk,cols=1),filename=paste(i, "_EId_", LookUpTable[i,1],"_CSID_",LookUpTable[i,4],

"_Quadriplot",".png"), width=floor(lastDay+2), height=15, units="in")

}

setwd("SOURCE")

write.csv2(PersonOfInterest, file ="ConvenienceSample.csv", row.names = FALSE)

#pati$day<-as.numeric(pati$day)

#singlePatient <- subset(pati, pati$day == DAY)

#table(pati$day)

#table(singlePatient$peep_m)

EventsAndDeviceDays.R

#

library(tidyverse)

library(lubridate)

library(plyr)

library(dplyr)

library(readr)

library(ggplot2)

source("SOURCE")

setwd("SOURCE")

#imports all relevant Datasets from the wd

CalculationSets <- read_delim("CalculationSets.csv", delim = ";")

Events <- read_delim("Events.csv", delim = ";")

#

# The same but this time the data is being ordered for Year

#

#

#

#

Intubationday <- subset(CalculationSets, CalculationSets$IsIntubatedAtNoon == "WAHR")

Intubationday$Date <- as.Date(Intubationday$Date, format = "%d.%m.%Y")

Intubationday$Year <- format(as.POSIXct(Intubationday$Date,format="%Y-%m-%d"),"%Y")

Intubationday <- as.data.frame(table(Intubationday["Year"]))

VentilatorEvents <- Events

VentilatorEvents$Date<- as.Date(VentilatorEvents$Date, format = "%d.%m.%Y")

VentilatorEvents$Year <- format(as.POSIXct(VentilatorEvents$Date,format="%Y-%m-%d"),"%Y")

VentilatorEvents <- as.data.frame(table(VentilatorEvents["Year"]),stringsAsFactors=FALSE)

VentilatorEvents <- merge(VentilatorEvents,Intubationday, by.x = "Var1", by.y = "Var1")

colnames(VentilatorEvents) <- c("Year","VAE","DeviceDays")

VentilatorEvents$Ratio <- VentilatorEvents$VAE/VentilatorEvents$DeviceDays*1000

ggplot(VentilatorEvents, aes(x=VentilatorEvents$Year, y= VentilatorEvents$Ratio))+

geom_point()+

scale_y_continuous(limits = c(0, 40), name="VAE / 1000 Device Days")+

scale_x_discrete(name="Year")

#ggplot(VentilatorEvents, aes(VentilatorEvents$Year, VentilatorEvents$Ratio)) +

# geom_point() +

# geom_smooth()

VentilatorEvents$Year<- as.numeric(VentilatorEvents$Year)

pois<-glm(VAE~Year+offset(log(DeviceDays)), data=VentilatorEvents, family=poisson)

exp(-0.032)

summary(pois)

print(cbind(exp(coef(pois)), exp(confint(pois)), coef(summary(pois))[,'Pr(>|z|)']))

exp(cbind(OR = coef(pois), confint(pois)))

exp(coef(pois))

ggplot(VentilatorEvents, aes(x=VentilatorEvents$Year, y= VentilatorEvents$Ratio))+

geom_smooth(method = "lm", se = FALSE, color = "gray80")

#BEstimmung CI für jedes Jahr

for (i in 1:9) {

p<-prop.test(VentilatorEvents[i,2], VentilatorEvents[i,3])

VentilatorEvents[i,5]<-p$conf.int[1]*1000

VentilatorEvents[i,6]<-p$conf.int[2]*1000

}

VentilatorEvents$Year<-factor(VentilatorEvents$Year)

ggplot(VentilatorEvents, x=VentilatorEvents$Year)+

geom_errorbar(aes(x=VentilatorEvents$Year, ymin=VentilatorEvents$V5, ymax=VentilatorEvents$V6), width=.2,

position=position_dodge(0.05))+

geom_point(aes(x=VentilatorEvents$Year, y=VentilatorEvents$Ratio), size=3)+

scale_y_continuous(limits = c(0, 40), name="VAE / 1000 Device Days")+

scale_x_discrete(name="Year")+

scale_size(range = c(0, 10))

wayne1 <- function(CalculationSets, Events, Month, Ratio, Year, mean, sd, count, year, denomit, dat, poisson) {

#

#

#

# Third version, we use the yearly mean but add the monthly values for standard deviation

#

#

#

Intubationday <- subset(CalculationSets, CalculationSets$IsIntubatedAtNoon == "WAHR")

Intubationday$Date <- as.Date(Intubationday$Date, format = "%d.%m.%Y")

Intubationday$YearMonth <- format(as.POSIXct(Intubationday$Date,format="%Y-%m-%d"),"%Y-%m")

Intubationday <- as.data.frame(table(Intubationday["YearMonth"]))

VentilatorEvents <- Events

VentilatorEvents$Date<- as.Date(VentilatorEvents$Date, format = "%d.%m.%Y")

VentilatorEvents$YearMonth <- format(as.POSIXct(VentilatorEvents$Date,format="%Y-%m-%d"),"%Y-%m")

VentilatorEvents <- as.data.frame(table(VentilatorEvents["YearMonth"]), stringsAsFactors=FALSE)

VentilatorEvents <- merge(VentilatorEvents,Intubationday, by.x = "Var1", by.y = "Var1")

VentilatorEvents$Var1 <- as.Date(paste(VentilatorEvents$Var1,"-15", sep = ""), format = "%Y-%m-%d")

colnames(VentilatorEvents) <- c("Month","VAE","DeviceDays")

VentilatorEvents$Ratio <- VentilatorEvents$VAE/VentilatorEvents$DeviceDays*1000

YearlyMeanAndSD <- subset(VentilatorEvents, select=c(Month, Ratio))

YearlyMeanAndSD$Year <- format(as.POSIXct(YearlyMeanAndSD$Month,format="%Y-%m-%d"),"%Y")

YearlyMeanAndSD$Month <-NULL

#a<-aggregate(YearlyMeanAndSD, list(YearlyMeanAndSD$Year), function(x) c(mean = mean(x), sd = sd(x)))

a<-YearlyMeanAndSD %>% group_by(Year) %>% summarise_each(funs(mean, sd))

a$plusSD <- a$mean+a$sd

a$minSD <- a$mean-a$sd

a$Year <- as.Date(a$Year, format="%Y")

#Version mit Konfidenzintervall um die Smooth-FUnktion

ggplot(a, aes(x=a$Year, y= a$mean))+

geom_smooth(size=1)+

scale_y_continuous(name="VAE / 1000 Device Days", limits = c(0,4))+

scale_x_date(name="Year")

#Version ohne, dafür aber mit der Standardabweichung der Ratio

ggplot(a, aes(x=a$Year, y= a$mean))+

geom_smooth(size=1, se=FALSE)+

geom_ribbon(data=a, ymin=a$minSD, ymax=a$plusSD, alpha=0.5, colour="blue", fill="blue")+

scale_y_continuous(name="VAE / 100 Device Days", limits = c(0,4))+

scale_x_date(name="Year")

#

glm(count~year+ offset(denomit), data=dat, family=poisson)

}

wayne2 <- function(CalculationSets, Events) {

# the df intubationday selects for isintubatedatnoon == TRUE

# converting the date format to an actual date

# extracting YearMonth

# ordering the data for YearMonth

Intubationday <- subset(CalculationSets, CalculationSets$IsIntubatedAtNoon == "WAHR")

Intubationday$Date <- as.Date(Intubationday$Date, format = "%d.%m.%Y")

Intubationday$YearMonth <- format(as.POSIXct(Intubationday$Date,format="%Y-%m-%d"),"%Y-%m")

Intubationday <- as.data.frame(table(Intubationday["YearMonth"]))

VentilatorEvents <- Events

VentilatorEvents$Date<- as.Date(VentilatorEvents$Date, format = "%d.%m.%Y")

VentilatorEvents$YearMonth <- format(as.POSIXct(VentilatorEvents$Date,format="%Y-%m-%d"),"%Y-%m")

VentilatorEvents <- as.data.frame(table(VentilatorEvents["YearMonth"]), stringsAsFactors=FALSE)

VentilatorEvents <- merge(VentilatorEvents,Intubationday, by.x = "Var1", by.y = "Var1")

VentilatorEvents$Var1 <- as.Date(paste(VentilatorEvents$Var1,"-15", sep = ""), format = "%Y-%m-%d")

colnames(VentilatorEvents) <- c("Month","VAE","DeviceDays")

VentilatorEvents$Ratio <- VentilatorEvents$VAE/VentilatorEvents$DeviceDays*1000

ggplot(VentilatorEvents, aes(x=VentilatorEvents$Month, y= VentilatorEvents$Ratio))+

geom_point(size=1)+

scale_y_continuous(name="VAE / 1000 Device Days", limits = c(0,4))+

scale_x_date(name="Month")+

geom_smooth(method = "lm", se = FALSE, color = "gray80")

}

totalEvents <- NA

totalEvents <-as.data.frame(totalEvents)

totalEvents$Events <- sum(VentilatorEvents$VAE)

totalEvents$DeviceDays <- sum(VentilatorEvents$DeviceDays)

totalEvents$Ratio <- totalEvents$Events/totalEvents$DeviceDays*1000

p<-prop.test(VentilatorEvents[1,2], VentilatorEvents[i,4])

VentilatorEvents[1,5]<-p$conf.int[1]*1000

VentilatorEvents[1,6]<-p$conf.int[2]*1000

EventsPerHEpisodes_export.R

library(tidyverse)

library(lubridate)

library(plyr)

library(dplyr)

library(readr)

library(ggplot2)

library(xlsx)

source("SOURCE")

setwd("SOURCE")

#imports all relevant Datasets from the wd

CalculationSets <- read_delim("CalculationSets.csv", delim = ",")

CalculationSets$Date <- as.Date(CalculationSets$Date,"%d.%m.%y")

Events <- read_delim("Events.csv", delim = ",")

VAEPerYear <- subset(Events, select = c("Date"))

VAEPerYear$Year <- year(as.Date(VAEPerYear$Date, "%d.%m.%Y"))

VAEPerYear <- as.data.frame(table(VAEPerYear["Year"]))

colnames(VAEPerYear) <- c("Year", "VAE")

#create a list of patients and calculate the number of ventilation episodes per year

setwd("SOURCE")

nativeData <- read_delim("vap_ventilated.csv", delim = ",")

nativeData$DateTime <- as.Date(nativeData$DateTime,"%y-%m-%d")

aggTable <- subset(nativeData, select = c("PatientID","DateTime"))

colnames(aggTable) <- c("PatientID","Year")

aggTable$Year <- year(aggTable$Year)

aggTable <- aggregate(aggTable$Year, by=list(aggTable$PatientID), FUN=min)

#VentilationEpisodesPerYear <- aggregate(aggTable

VentilationEpisodesPerYear <- as.data.frame(table(aggTable["x"]))

colnames(VentilationEpisodesPerYear) <- c("Year", "VentilationEpisodes")

VentilationEpisodesPerYear <- merge(VentilationEpisodesPerYear,VAEPerYear, by.x = "Year", by.y = "Year")

VentilationEpisodesPerYear$IncidencePerHundred <- VentilationEpisodesPerYear$VAE*100/VentilationEpisodesPerYear$VentilationEpisodes

for (i in 1:9) {

p<-prop.test(x = VentilationEpisodesPerYear[i,3], n = VentilationEpisodesPerYear[i,2])

VentilationEpisodesPerYear[i,5]<-p$conf.int[1]*100

VentilationEpisodesPerYear[i,6]<-p$conf.int[2]*100

}

write.xlsx("SOURCE")

ggplot(VentilationEpisodesPerYear, x=VentilationEpisodesPerYear$Year)+

geom_point(aes(x=VentilationEpisodesPerYear$Year, y=VentilationEpisodesPerYear$IncidencePerHundred), size=3)+

geom_errorbar(aes(x=VentilationEpisodesPerYear$Year, ymin=VentilationEpisodesPerYear$V5, ymax=VentilationEpisodesPerYear$V6), width=.2,

position=position_dodge(0.05))+

scale_y_continuous(limits = c(0, 8), name="VAE / 100 Ventilation Episodes")+

scale_x_discrete(name="Year")+

scale_size(range = c(0, 5))

ValidateIVAC.R

library(tidyverse)

library(lubridate)

library(dplyr)

library(readr)

library(ggplot2)

library(plyr)

source("SOURCE")

setwd("SOURCE")

#imports all relevant Datasets from the wd

CalculationSets <- read_delim("CalculationSets.csv", delim = ";")

Events <- read_delim("Events.csv", delim = ";")

Medication <- read_delim("Medications.csv", delim = ";")

Patients <- read_delim("Patients.csv", delim = ";")

#set maximum and minimum VAE-Window boundaries

# Pseudorandom sample

set.seed(261018)

sEvents <- sample_n(Events,100)

sEvents <- merge(sEvents, Patients, by.x = "Patient_Id", by.y = "Id", all.x = TRUE)

VAEWindow <- subset(sEvents, select=c(Date, Patient_Id))

VAEWindow$MinDay <- as.numeric(as.Date(VAEWindow$Date, format = "%d.%m.%Y") -2)

VAEWindow$MaxDay <- as.numeric(as.Date(VAEWindow$Date, format = "%d.%m.%Y") +2)

i <- subset(Medication, select=c(Patient_Id,Start))

i<- merge(i, VAEWindow, by.x = "Patient_Id", by.y = "Patient_Id")

i$Start <- as.numeric(as.Date(i$Start, format = "%d.%m.%Y"))

i$Flag <- FALSE

i$Flag[i$Start>=i$MinDay & i$Start<=i$MaxDay] <- TRUE

noIVAC <- subset(i, i$Flag==FALSE)

noIVAC <- subset(noIVAC, select=c(Patient_Id, Date, Flag))

IVAC <- subset(i, i$Flag==TRUE)

IVAC <- subset(IVAC, select=c(Patient_Id, Date, Flag))

Results <- rbind(unique(IVAC),unique(noIVAC))

Results <- Results[order(Results$Patient_Id),]

sEvents <- subset(sEvents, select=c(Date, Patient_Id, Type))

Results<- merge(Results, sEvents)

Results$IVAC <- FALSE

Results$IVAC[Results$Type==1]<-TRUE

MissingMed <- read_delim("MissingMedication.csv", delim = ";")

MissingMed <- merge(MissingMed, sEvents)

#i <- subset(i, i$Start>=i$MinDay)

#i <- subset(i, i$Start<=i$MaxDay)

#i$Date<-as.numeric(as.Date(i$Date, format = "%d.%m.%Y"))

#i$Flag <- 1

#IVACChart <- subset(Events, select = c(Patient_Id, Date, Type))

#IVACChart$Date <- as.numeric(as.Date(IVACChart$Date, format = "%d.%m.%Y"))

#IVACChart <- merge(IVACChart,i, all.x = TRUE)

#SinglePatient <- subset(CalculationSets, Patient_Id == ID)

#IVACFails <- subset(IVACChart, is.na(IVACChart$MinDay))

#i$Flag <- as.numeric(i$Start) >= as.numeric(i$MinDay)

#by.x =c ("Patient_Id", "Date"), by.y = c("Patient_Id","Date")
